# Lipidomics Reveals That Rice or Flour as a Single Source of Carbohydrates Cause Adverse Health Effects in Rats

**DOI:** 10.3389/fnut.2022.887757

**Published:** 2022-05-20

**Authors:** Siyu Wang, Wenjun Wang, Hongmei Mao, Mingyu Zhu, Zihan Xu, Jun Wang, Xuesong Zhang, Baolong Li, Xuesong Xiang, Zhu Wang

**Affiliations:** ^1^Key Laboratory of Trace Element Nutrition of National Health Commission, National Institute for Nutrition and Health, Chinese Center for Diseases Control and Prevention, Beijing, China; ^2^Beijing Junfeix Technology Co., Ltd., Beijing, China; ^3^Shenzhen Polytechnic, School of Food and Drug, Shenzhen, China

**Keywords:** rice diet, wheat flour diet, AIN-93 diet, lipidomics, triacylglycerols

## Abstract

The type of diet is very important for the maintenance of health and nutrition. How the sole source of carbohydrates from rice- or flour-based diet affect blood sugar has not been elucidated for a long time. In order to explore the effects of these diets, sixty SD rats were randomly divided into three groups: control group (C group, AIN-93, standard diet), rice diet group (R group), and flour diet group (F group). All the rats were fed for 7 weeks in total by the assigned diets for 4 weeks (stage 1, S1) and all by the AIN-93 diet for 3 weeks (stage 2, S2). The body weights of all the rats were monitored and serum samples were taken for testing blood glucose, biochemical indicators and untargeted lipidome. It was found that both rice and flour-based diets caused weight gain, but the flour diet had a significant increase in blood sugar and low-density lipoprotein (LDL), while a significant decrease in albumin (ALB) and triglycerides (TG). Twenty-three and 148 lipids were changed by lipidomics in the rice diet group and flour diet group, respectively, and two lipids showed the same changes in the two groups, all belonging to TGs, namely TG (16:0/16:0/16:1) and TG (16:0/16:1/18:2), which showed that a single diet source had a significant effect on the health of rats. Fortunately, we can recover this effect through the subsequent standard diet, allowing the rats to return to normal blood sugar, weight and biochemical indicators. A model can predict the diet types through the logistic regression method. Finally, we proposed that a single diet increased blood sugar and weight through a decrease in TGs, and blood sugar and weight returned to normal after a standard diet. Taken together, the short-term negative effects caused by a single diet can be recovered by a standard diet and further proves the importance of diet types.

## Introduction

People intake different varieties of grains as the main source of carbohydrates, which may lead to different effects on health (1, 2), especially on blood glucose (3). In the north and south of China, the diets are significantly different with flour as the main grain in the north and rice in the south. Whether intake of a rice-based diet or flour-based diet will affect people's health remains unanswered.

At present, studies suggest that refined rice or wheat flour has adverse effects on human health, and diets based on these refined grains affect glucose tolerance (4). Most of these studies compared refined rice or flour with whole grains. A whole-grain-based diet is considered healthier because it is rich in crude fiber, vitamins, and trace elements (5, 6). Whole grains can significantly improve the blood glucose levels in people with type 2 diabetes (7). Meanwhile, whole-grain intake reduced LDL cholesterol, total cholesterol (TCHO), and triglyceride (TG) levels (8). Regarding the comparison between refined rice and flour, some studies have focused on factors related to obesity, such as a higher BMI in middle-aged and older men eating wheat-based foods compared with consuming rice (9). Evidence suggested that eating wheat was significantly positively associated with the prevalence of obesity and that eating rice was negatively associated with obesity (10). However, there is only a little research elucidating the differences in the effects between rice- and wheat flour-based diets, especially at the metabolic level.

Lipids are essential biomolecules that play a crucial role in human health. Driven by the significance of lipid biology, lipidomics has become an emerging technology in nutrition (11), especially in diet (12). The current lipidomics research in grains primarily focuses on whole grains. Lipidomics analysis showed that polyunsaturated fatty acid (PUFA) levels in lipids such as phosphatidylcholine (PC), PC-ether, and phosphatidylinositol were increased in the plasma of mice fed a whole grain, bran, and aleurone supplemental diet when compared to a diet of refined white flour (13). Treatment with rice bran, which partially reduced the high-energy diet-induced increases in DG (18:2/18:1) and TG (18:0/16:0/18:3) in obese rats, significantly reduced serum uric acid, glucose, liver triglycerides (TG), and total cholesterol (TCHO) levels (14). Diets rich in whole grains present a lower risk of disease than diets based on refined grains, which can modulate the intestinal microflora composition and increase short-chain fatty acids (SCFA) concentrations (15). Studies about WG consumption as advantageous to human health suggest that it is associated with a significantly lower risk of all-cause mortality (16). In our previous study, we found that long-term administration of refined grain leads to changes in the levels of fasting blood glucose and renal pathology, as well as dyslipidemia in rats. An appropriate administration of whole grains can delay the progression of adverse symptoms (17). In 2021, we explored the effects of whole rice, common feed, and refined rice on normal rats through non-targeted metabolomics. The fasting blood glucose level of the refined rice group was significantly higher than that of the brown rice group and the control group, and 12 potential differential metabolites were found. Higher consumption of refined rice may lead to increased blood glucose levels. Brown rice helps maintain relatively low blood lipid levels, and glycerophospholipid metabolism is a common pathway for rice to affect blood glucose and lipid metabolism in normal rats (18).

In our research we chose AIN-93 diet as the feed of the control group, AIN-93 diet is the purified diet proposed by the American Institute of Nutrition. Standardization of diets for laboratory animals could reduce the variation in the whole experiment and facilitate reproducibility in research (19). In the experiments on rats with cereals, AIN-93 diet was often used as the basic feed or the control feed (20). Therefore, we chose the AIN-93 diet as the control group's diet and the based diet for rice and wheat diet group. Here, we tested the effects of three diet types and fed rats at two stages and found that a single diet increased blood sugar and weight while a decrease in TGs, and blood sugar and weight returned to normal after a reference diet.

## Materials and Methods

### Chemicals and Reagents

Biochemistry analysis reagents were obtained from Hitachi Company (Tokyo, Japan). Leucine-enkephalin (LE) and sodium formate from Waters Company (Milford, MA, USA). All lipid standards and internal standards were purchased from Avanti Polar Lipids Inc. (Alabaster, AL, USA). Formic acid (≥95%), methanol, and isopropanol were all HPLC grade, obtained from Thermo Fisher Scientific (Waltham, MA, USA). Acetonitrile and ddH_2_O were all HPLC grade, obtained from Merck Company (Darmstadt, Germany).

### Rats and Feed

Healthy male SPF-grade Sprague-Dawley (SD) rats (28 days old, weighing 85–120 g each) were purchased from Beijing Vital River Laboratory Animal Technology Co., Ltd. (License No. SCXK (Jing) 2018-0006). The room temperature for animal feeding was kept between 18–22°C, relative humidity between 30–70%, with 12 h of light and 12 h of darkness daily. Rats were fed with three kinds of feed based on the experimental design. All three feeds were commissioned by the company for the product. The three different feeds were AIN-93G, milled rice formula feed, and milled flour formula feed. In the latter two diets, the corn starch and sucrose in the AIN-93G diets were replaced with milled rice and refined wheat flour, respectively. Both rice and wheat flour are sourced from brands commonly found in the market. Table 1 lists the formulation of the three groups' diets.

**Table 1:** Three groups of feed formula.

**Ingredient (g/kg)**	**Control group**	**Rice diet group**	**Flour diet** **group**
Casein	200	200	200
Cystine	3	3	3
Rice flour	0	630	0
Wheat flour	0	0	630
Corn starch	397	0	0
Maltodextrin	132	0	0
Sucrose	100	0	0
Cellulose	50	50	50
Soybean oil	70	70	70
Choline hydrogen tartrate	3.0	3.0	3.0
AIN mineral blend	35	35	35
AIN vitamin blend	10	10	10

### Experimental Design

**Figure 1:**
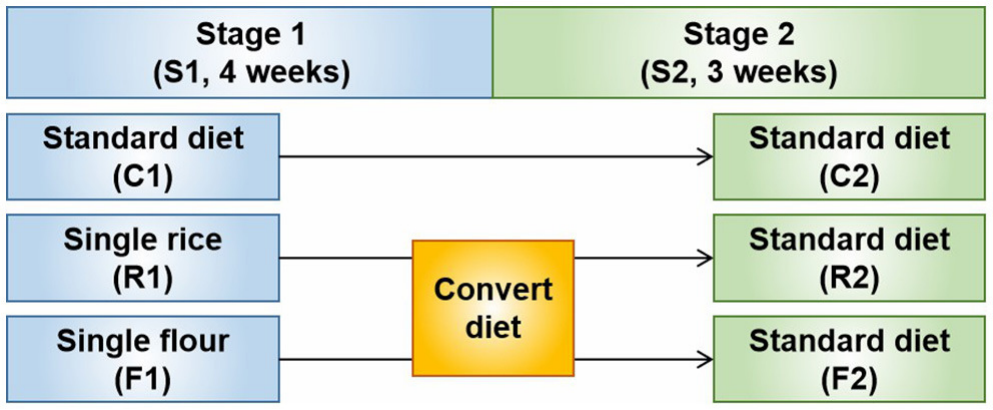
Schematic diagram of the experimental design. Stage 1 (S1) involved treatment with a rice (R1) and flour (F1) diets. After converting the diets at Stage 2 (S2), R1 and F1 became the AIN-93 diet (R2 and F2, respectively) for 3 weeks.

A schematic of the experimental design is shown in Figure 1. The animals were randomly assigned to three groups: control group (C), rice diet group (R), and flour diet group (F). Twenty rats were assigned to each group. Before the experiment, all the rats were fed the AIN-93 control rodent chow diet for 1 week. The experimental period was divided into two stages. In the first stage, the control group was fed the AIN-93 diet, the rice group was fed a milled rice formula diet, and the flour group was fed a milled wheat flour formula diet. This stage lasted for 4 weeks. In the second stage, the control, rice, and flour groups were fed the AIN-93 diet. This stage lasted for 3 weeks. The weight of the rats was measured twice a week throughout the animal experiment. The rats were fasted overnight for 12 h before the end of each stage and weighed. In the morning after fasting, a peritoneal injection of 2% pentobarbital sodium was used for anesthesia according to body weight, and blood was collected and prepared into serum and plasma samples. The samples were immediately frozen with liquid nitrogen and stored at −80°C.

### Blood Biochemistry Analysis

Total cholesterol (TCHO), triacylglycerol (TG), high-density lipoprotein (HDL), and low-density lipoprotein (LDL) levels in serum were measured using an automatic biochemical analyzer (Hitachi 7600-210E, Tokyo, Japan). All assays were performed according to the manufacturer's protocols.

### Lipidomics Analysis

Lipidomics measurement was conducted according to previous studies (21, 22). Lipid extraction was performed according to previously described methods (23, 24). The collected serum (100 μL) was used for lipid extraction. LPC (18:1) (d7) was used as the internal standard and all samples were aspirated 20 μL into the same vial as QC (quality control) samples. The sample vials were stored at −20°C until detection. Lipidomics analysis was performed using a UPLC system (ACQUITY UPLC *I*-Class, Waters, USA) coupled with an electrospray ionization quadrupole time-of-flight mass spectrometer (ESI-QTOF MS; SYNAPT G2-Si HDMS, Waters, USA). A Waters ACQUITY HSS T3 column (1.7 μm; 100 mm × 2.1 mm) was used for LC separation, and the column temperature was maintained at 40°C. The MS^E^ data were acquired in the continuum mode using the ramp collision energy in two scan functions.

### Data Analysis

Raw data were imported into the commercial software Progenesis QI (Version 2.4, Waters) for data processing and identification was performed using LIPIDMAPS database. Data with a relative reference deviation (RSD) of >30% in quality control (QC) samples were filtered. Partial least squares discriminant analysis (PLS-DA) was performed, and variable importance in projection (VIP) was calculated using MetaboAnalyst 5.0 (25). Column figures were drawn using GraphPad Prism 8.0 software. All data are expressed as the mean ± SE. Differences between groups were analyzed using the *t*-test. Differences were considered statistically significant at *p* < 0.05. Differential lipids were defined as *p* < 0.05 and VIP > 1.

## Results

### Weight and Blood Glucose

**Figure 2:**
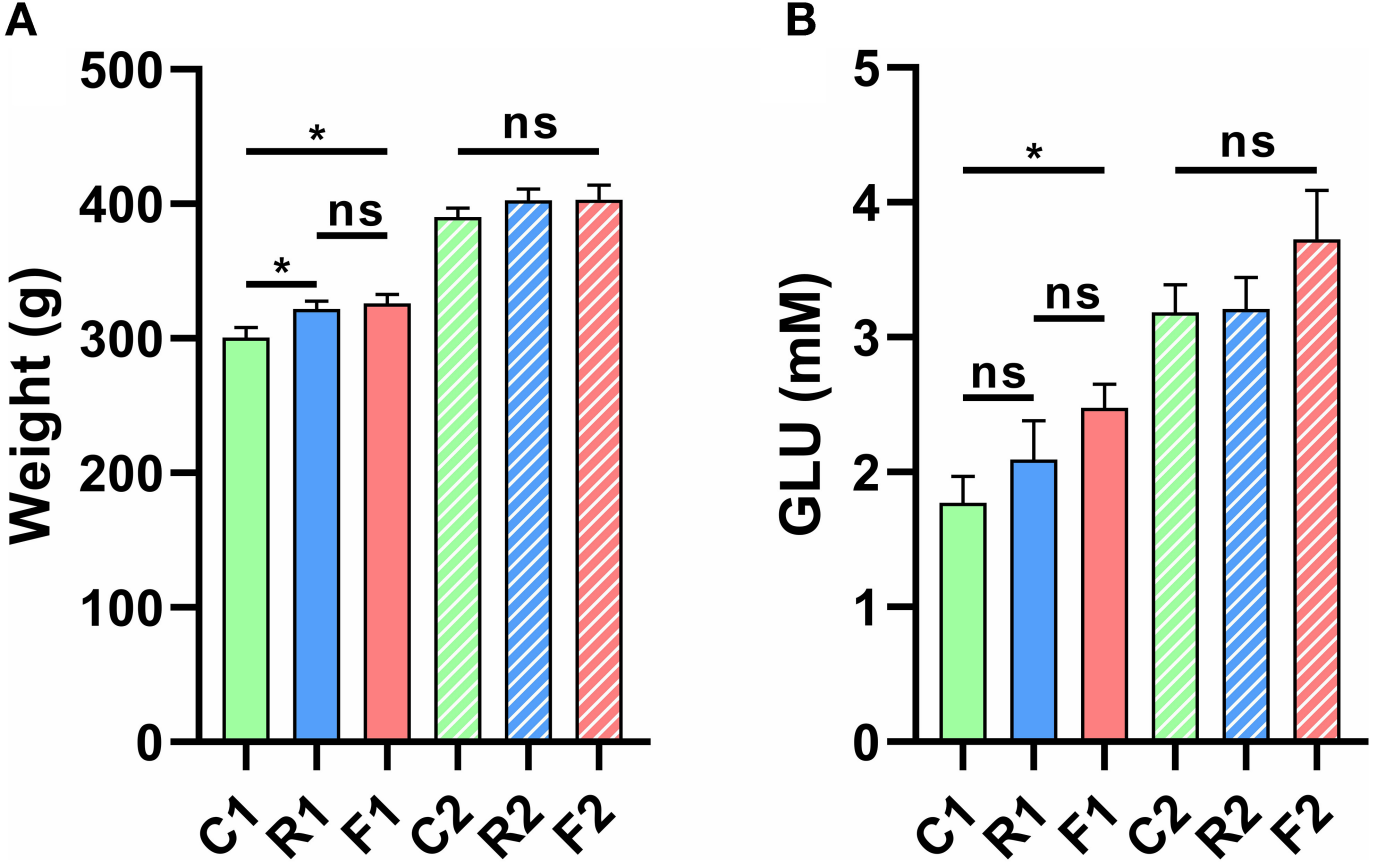
Changes in body weight and glucose (GLU) levels of rats fed different diets and at different experimental stages. **(A)** The body weights of rats were significantly increased in R1 vs. C1 and F1 vs. C1 at stage 1, but no significant differences were observed at stage 2. **(B)** Glucose levels were significantly increased in F1 vs. C1 at stage 1, but no significant differences were observed at stage 2. *p < 0.05; ns, not significant. *n* = 9–10 (R1 have 9 rats, C1, F1, C2, R2, and F2 have 10 rats). “n” means the number of samples/rats per group.

The results showed that rice and flour significantly increased body weight and blood glucose levels (*p* < 0.05) (Figure 2A). However, body weight and blood glucose levels returned to normal after the rats were converted to an AIN-93 diet for three weeks (Figure 2B).

### Biochemical Indicators

**Figure 3:**
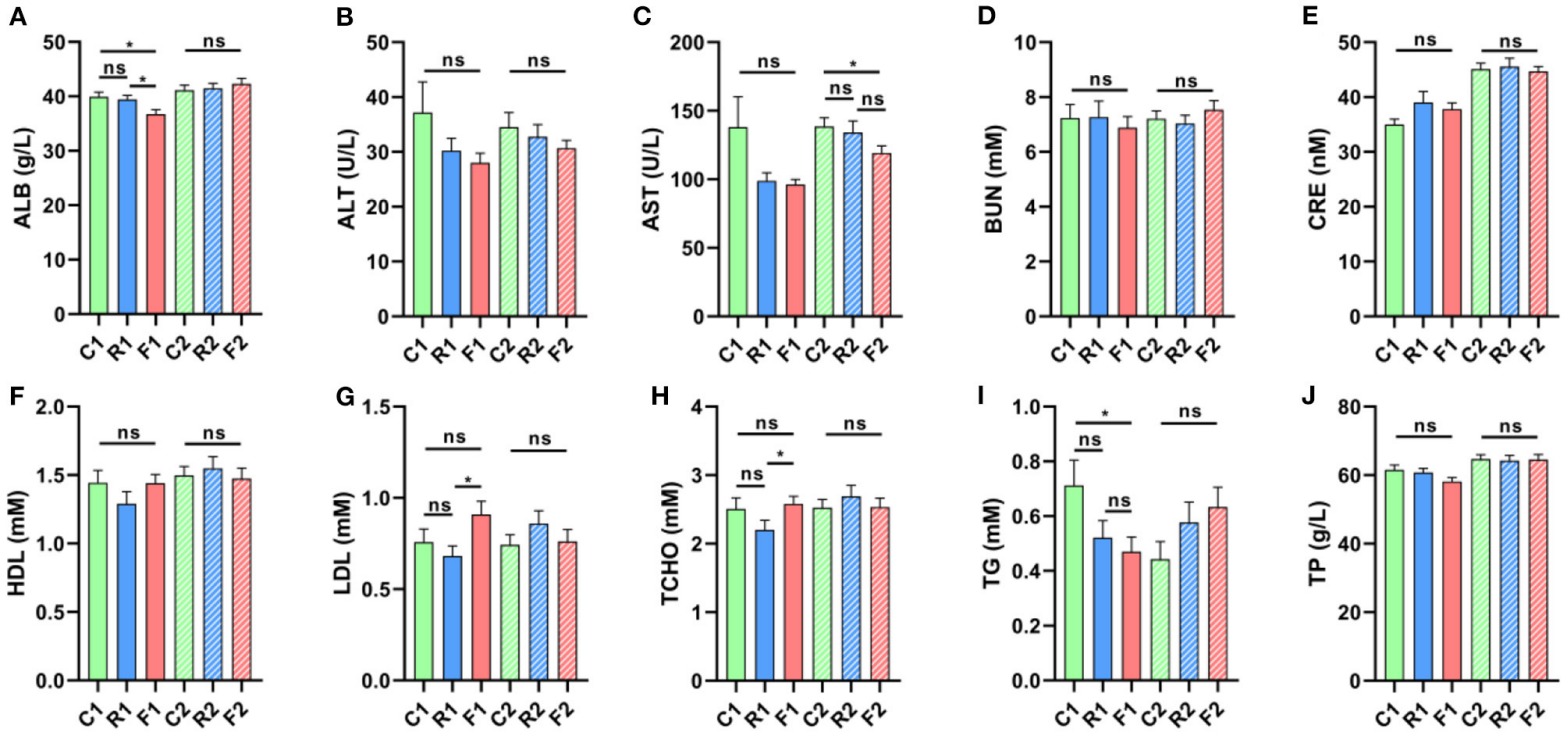
Changes in blood biochemistry indicators in different types of diets and stages. **(A)** ALB had a significant decrease, F1 vs. C1 and F1 vs. R1 at stage 1, but not a significant difference at stage 2. **(B)** ALT did not change significantly at stage 1 and 2. **(C)** AST showed no significant change at stage 1, but had a significant decrease at stage 2, F2 vs. C2. **(D, E, F)** BUN, CRE, HDL are not a significant at stage 1 and 2, respectively. **(G)** LDL has a significant increase, F1 vs. R1 at stage 1, but not a significant difference at stage 2. **(H)** TCHO has significant increase, F1 vs. R1 at stage 1, but has not a significant difference at stage 2. **(I)** TG has significant decrease, F1 vs. C1 at stage 1, but not a significant difference at stage 2. **(J)** TP has not a significant at stages 1 and 2. *is presented *p* < 0.05, ns is no significant. *n* = 9–10. (R1 have 9 rats, C1, F1, C2, R2, and F2 have 10 rats). “*n*” means the number of samples/rats in per group.

Blood biochemical indicators could reflect the physiological characteristics of organisms. In different diets and treatment stages of rats, we found that some indicators changed significantly, including ALB, AST, LDL, TCHO, and TG, but most indicators did not change at stage 1, which indicated that the rice and flour diet did not cause significant changes in physiological and biochemical levels. Fortunately, ALB, LDL, TCHO, and TG can recover to normal at stage 2, but the difference in AST only at stage 2 (Figure 3). The results showed that dietary differences had little effect on physiology and biochemistry and the differences caused by a single diet (stage 1) could be recovered by an AIN-93 diet (stage 2).

### Chromatogram and PCA Scores Plot of Lipidomics Data

**Figure 4:**
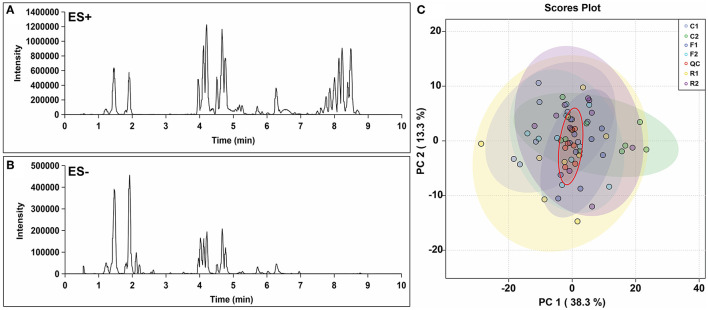
Chromatogram and PCA score plot of lipidomics data. **(A)** Lipidomic chromatogram at positive ion mode (ES+). **(B)** Lipidomic chromatogram at negative ion mode (ES-). **(C)** PCA score plot from the lipidomics data for all samples, including quality control (QC). *n* = 9-10. (R1 have 9 rats, C1, F1, C2, R2, and F2 have 10 rats). “n” means the number of samples/rats per group.

Over 10,000 peaks (the combination of positive and negative ion modes) were obtained through non-targeted lipidomics, and 514 quantitative and qualitative lipids were obtained through the identification and filtration of data with large deviations. Chromatograms were obtained in the positive ion mode (ES+) and negative ion mode (ES-) are shown in Figures 4A,B. PCA analysis of the 514 lipids showed that the QC group could be well aggregated in the middle of all samples, indicating that the quality control and stability of the experiment were reliable (Figure 4C).

### PLS-DA Model and Distribution of Differential Lipids

**Figure 5:**
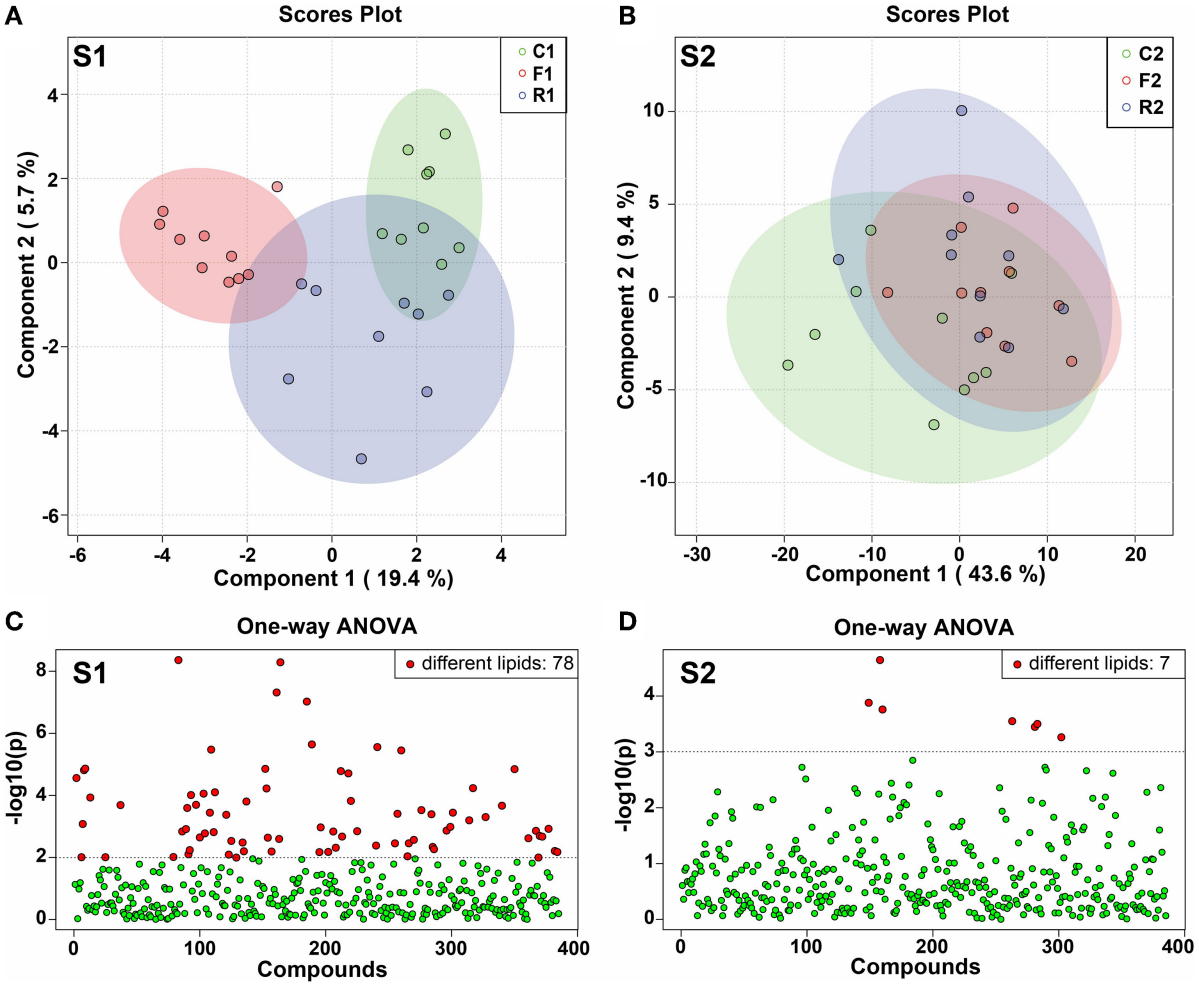
PLS-DA score plot and distribution of the differential lipids. **(A)** PLS-DA score plot at stage 1 (S1). **(B)** PLS-DA score plot at stage 2 (S1). **(C)** Distribution of differential lipids from one-way ANOVA analysis at stage 1 (S1), showing 78 different lipids. **(D)** Distribution of differential lipids using one-way ANOVA analysis at stage 2 (S2), showing 7 different lipids. *n* = 9–10. (R1 have 9 rats, C1, F1, C2, R2, and F2 have 10 rats). “*n*” means the number of samples/rats per group.

The PLS-DA model is a supervised multivariate statistical analysis method. We used PLS-DA for model analysis in the two experimental stages. At stage 1, there was a significant difference among the three groups. R1 and C1 were close to each other, but F1 and C1 were completely separated, and the difference was significant (Figure 5A). At stage 2, the three groups almost coincided, without a significant difference (Figure 5B). In addition, one-way ANOVA was performed on the 514 lipids, and the p-value was plotted. The results showed that at stage 1, 78 lipids were different among the three groups (Figure 5C), while at stage 2, seven lipids were different among the three groups (Figure 5D). The results indicated that at stage 1, there was a significant difference among the three groups. However, after the diet was converted, the difference among the three groups disappeared and returned to the normal level.

### Lipid Classification Showing That TGs Are the Main Lipids

**Figure 6:**
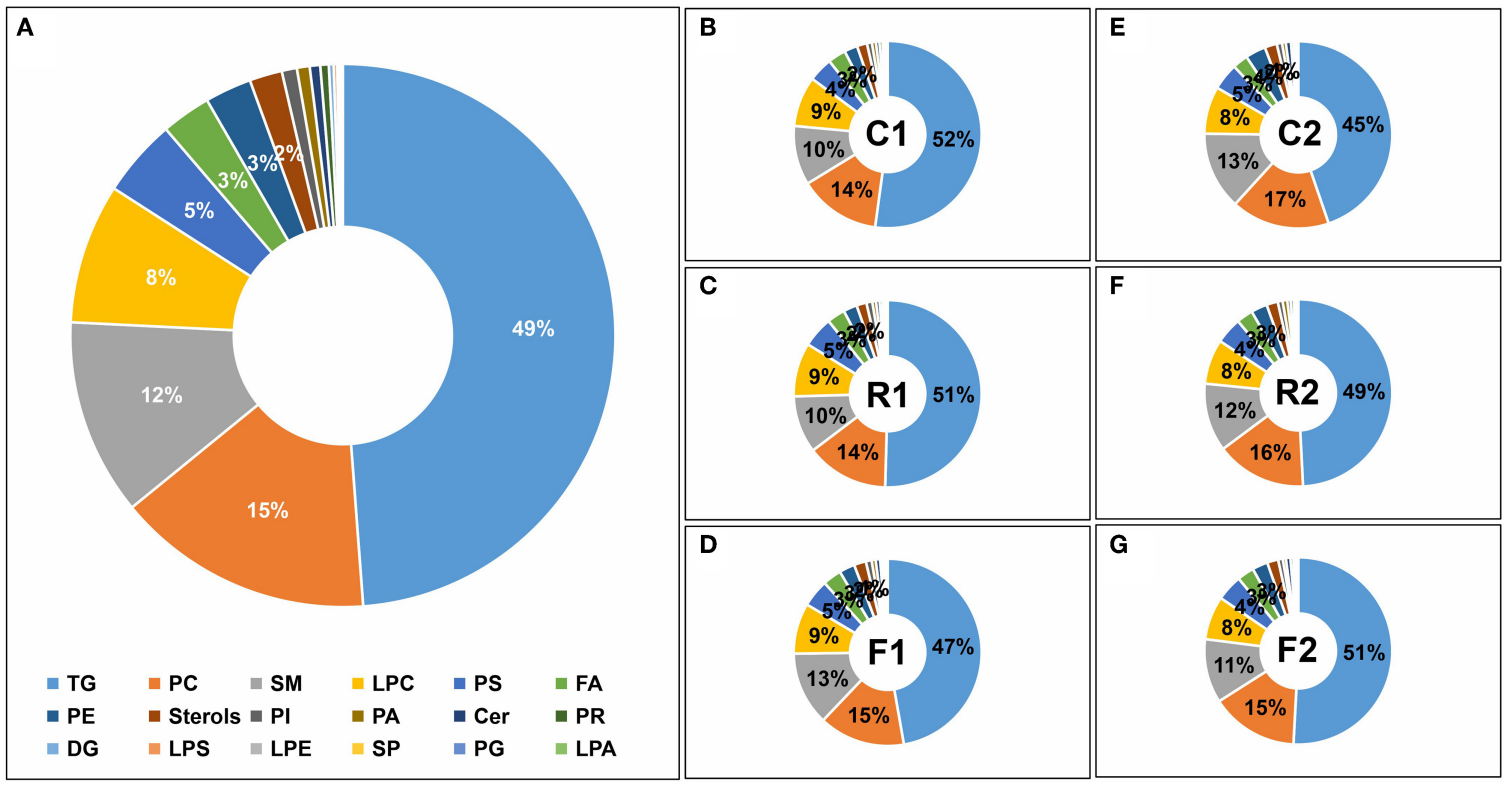
Lipid classifications of the different diet types. Lipids were classified into 18 categories, including triacylglycerol (TG), phosphatidylcholine (PC), sphingomyelin (SM), lysoPC (LPC), phosphatidylserine (PS), fatty acids (FA), phosphatidylethanolamine (PE), Sterols, phosphatidylinositol (PI), phosphatidic acid (PA), ceramides (Cer), prenol lipids (PR), diacylglycerol (DG), lysoPS (LPS), lysoPE (LPE), sphingolipids (SP), phosphatidylglycerol (PG), and lysoPA (LPA). **(A)** Pie graph of the lipid classifications from the average of all samples. **(B)** Pie graph of lipid classifications from the control group at stage 1 (C1). **(C)** Pie graph of lipid classifications from the rice diet group at stage (R1). **(D)** Pie graph of lipid classifications from the flour diet at stage 1 (F1). **(E)** Pie graph of lipid classifications from the control group at stage 2 (C2). **(F)** Pie graph of lipid classifications from the rice group at stage 2 (R2). **(G)** Pie graph of lipid classifications from the flour group at stage 2 (F2). Note that numbers are not displayed for lipid percentages below 3%.

According to the lipid classification database Lipidmaps (26) (https://www.lipidmaps.org/), the 514 lipids were divided into 18 categories, with the top seven categories accounting for more than 95% of the lipids; TGs accounted for approximately 50% (Figure 6A). Among them, TG levels decreased after rice and flour treatment but increased when the mixed diet was restored, with little change in the proportions of other lipids. Lipid classification not only indicated the lipid composition of rat serum during the diet but also indicated that F1 TGs decreased significantly after a single rice diet and were able to return to normal levels after switching to the AIN-93 diet (Figures 6B–G).

### Venn Diagram and Key Differential Lipids

**Figure 7:**
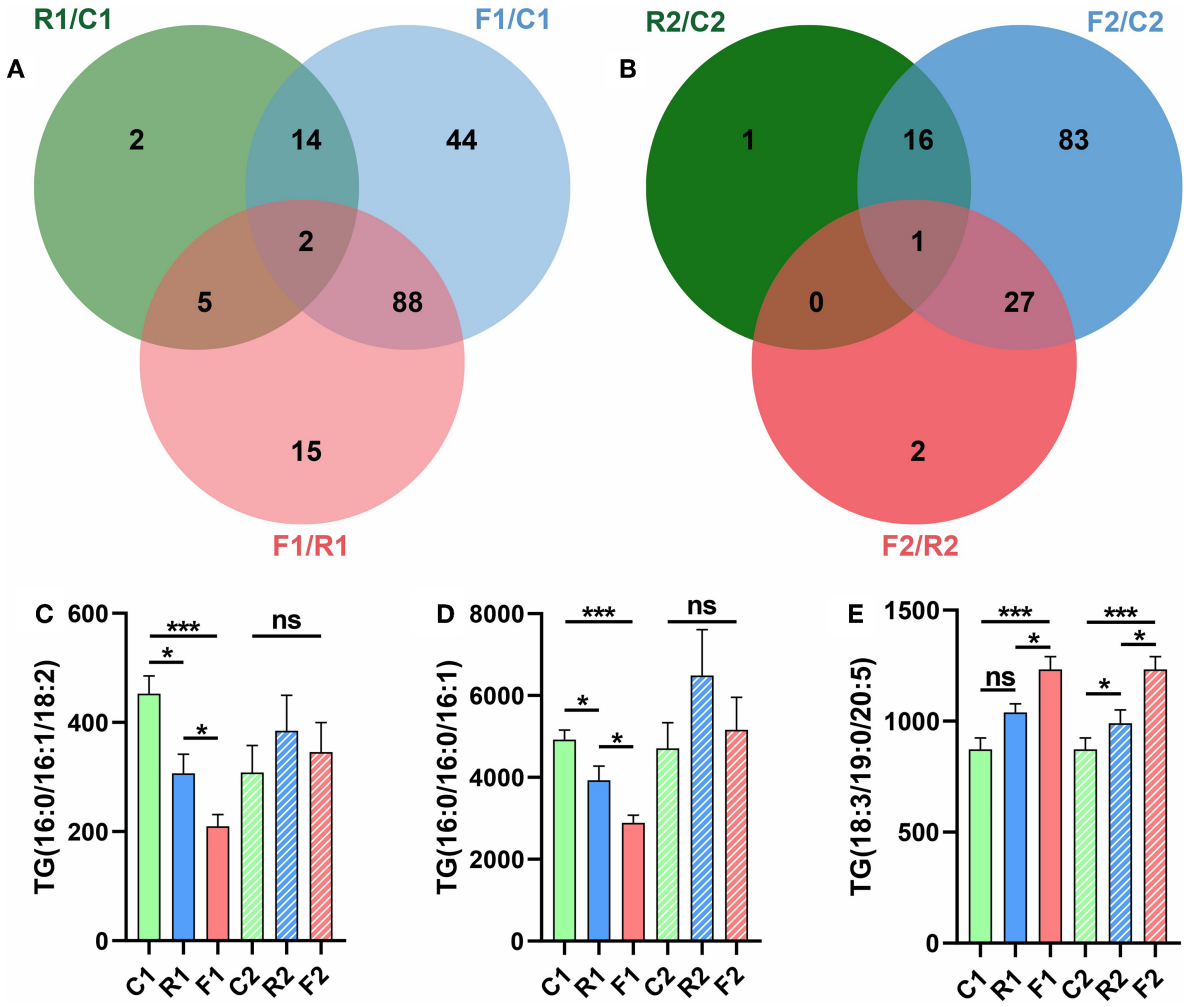
Venn diagram showing that TGs are the key differential lipids. **(A)** Pairwise comparisons were made among the three groups at stage 1, and two of the three comparisons had identical differential lipids. **(B)** Pairwise comparisons were made among the three groups at stage 2, and one of the three comparisons showed identical differential lipids. **(C)** Column diagram of the differential lipid TG (16:0/16:0/18:2). **(D)** Column diagram of the differential lipid TG (16:0/16:0/16:1). **(E)** Column diagram of the differential lipid TG (18:3/19:0/20:5). *p < 0.05; ***P < 0.001; ns, not significant. *n* = 9–10 (R1 have 9 rats, C1, F1, C2, R2, and F2 have 10 rats). “*n*” means the number of samples/rats per group.

We next obtained the VIP value through the PLS-DA model and selected lipids with VIP > 1 and *p* < 0.05 as the differential lipids. The rice diet alone showed seven differential lipids; the flour diet alone, 132 lipids; the rice and flour diets together, 16 lipids (Figure 7A). The recoverable difference of the rice diet group was as follows: one lipid; at stage 1, the three groups had two lipids in common: TG (16:0/16:1/18:2) and TG (16:0/16:0/16:1), which completely returned to the normal level after conversion to the AIN-93 diet (Figures 7C,D). The recoverable difference in the flour-fed group was 110 lipids, while that between the rice and flour diet group was 17 lipids. After the restoration with the AIN-93 diet, the three groups had one lipid in common, TG (18:3/19:0/20:5) (Figure 7E). The lipid profiles could not be fully recovered after being converted into the AIN-93 diet; in particular, the flour diet group showed a significant difference (Figure 7B). The results showed that diet types can significantly change lipid levels and that the AIN-93 diet can return the lipid profiles to normal. Furthermore, TGs are key lipids that may serve as biomarkers for diet types.

### TGs Were Correlated With GLU Levels in the Flour Diets

**Figure 8:**
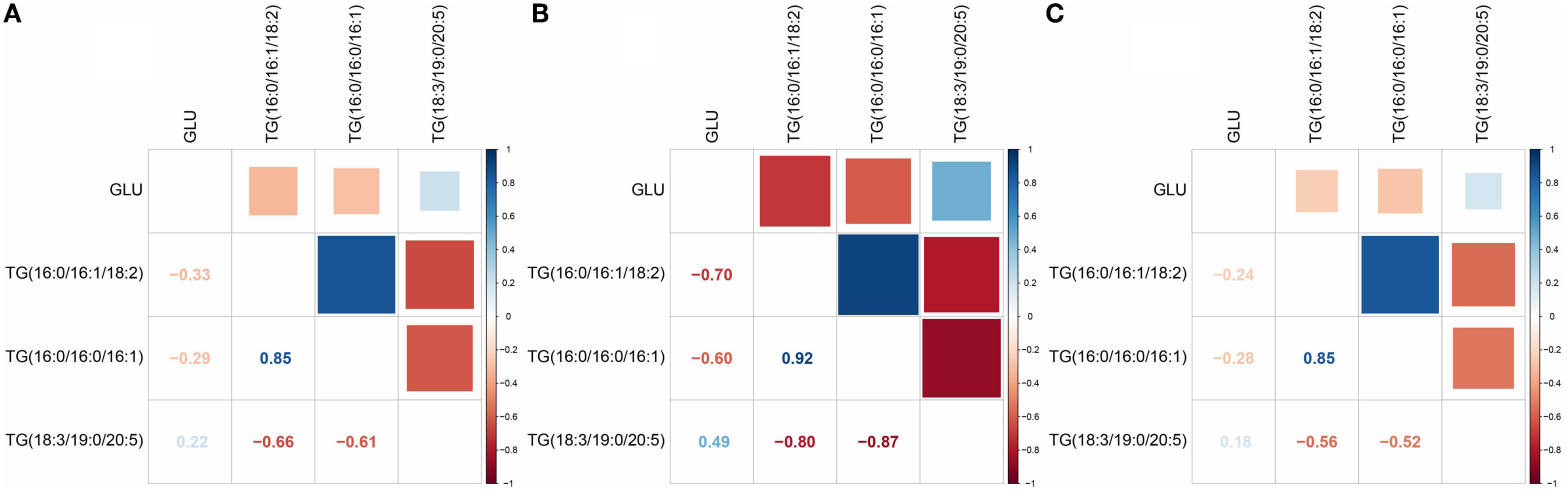
Correlation analysis between TGs and blood glucose (GLU). **(A)** Correlation analysis of the rice diet group at stage 1 (R1) vs. the control group at stage 1 (C1). **(B)** Correlation analysis of the flour diet group at stage 1 (F1) vs. C1 at stage 1. **(C)** Correlation analysis of the rice diet group at stage 2 (R2) vs. the control group at stage 2 (C2). The figures in the picture refer to the correlation coefficient. The positive value of blue is positively correlated, and the negative value of red is negatively correlated.

We used Pearson correlation analysis to identify the key relationship between differential lipid TGs and blood glucose levels. The results showed that in the rice diet, the correlation between TGs and blood glucose was very low, with the correlation coefficient up to 0.33 (Figure 8A). However, the correlation between TGs and blood glucose was stronger in the flour diet, with the correlation coefficient up to 0.7 (Figure 8B). The correlation between TGs and blood glucose was very low in the rice diet compared with the flour diet (Figure 8C). The different values indicated that TGs had a better correlation with blood glucose in the flour diet, which might be the main reason for the increased blood glucose caused by the flour-fed diet. TGs might be an induction factor for the increase in blood glucose level.

### Logistic Regression Model Showing That TGs Could Predict Diet Type

**Figure 9:**
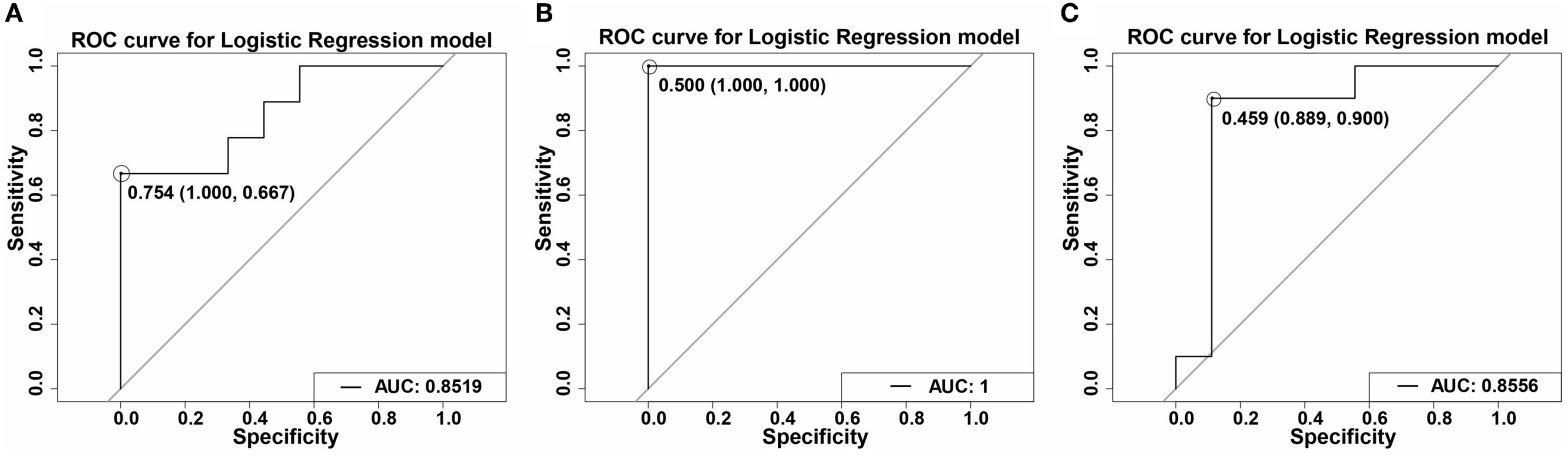
ROC curves of the TGs using a logistic regression model. **(A)** ROC curve of R1 vs. C1 of the TGs using a logistic regression model at stage 1. AUC = 0.8519. **(B)** ROC curve of F1 vs. C1 of the TGs using a logistic regression model at stage 1. AUC = 1. **(C)** ROC curve of R2 vs. C2 of the TGs using a logistic regression model at stage 1. AUC = 0.8556. TGs including TG (16:0/16:1/18:2) and TG (16:0/16:0/16:1). The figures in the picture refer to the cutoff values. The numbers in the parentheses are the sensitivity and specificity, respectively.

TGs, including TG (16:0/16:0/18:2) and TG (16:0/16:0/16:1), are the key differential lipids in the different diets administered in this study. We used these two TGs to establish a model using the logistic regression method and evaluated using the ROC curve. We obtained good AUC values of 0.8519, 1, and 0.8556 in the rice diet and AIN-93 diet, the flour diet and AIN-93 diet, and the rice and flour diets, respectively (Figures 9A–C). In addition, TGs have good sensitivity and specificity, especially in the flour diet and AIN-93 diet, which achieved 100% specificity and sensitivity. These results indicated that the model established by the two TGs could determine the type of diet.

### Diet Types Affect Body Weight and Blood Glucose by Regulating TGs

**Figure 10:**
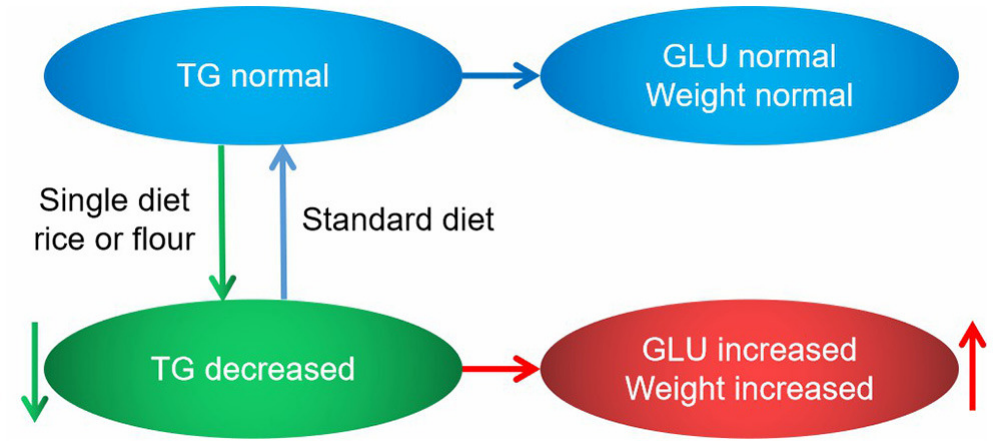
Diagram of the mechanism showing how diet types affect body weight and blood glucose levels. Blue ellipses and lines are presented as normal states, green ellipses and lines are presented as normal states, and red ellipses and lines are presented as normal states.

Based on the previous analysis, we clarified that different diet types affected body weights and blood glucose levels. Rice and flour diets alone can significantly increase body weights, while the flour diet alone can significantly increase blood glucose levels. The rice diet also caused a slight increase in blood glucose, but the increase was not significant (Figure 10). It was found from the lipidomics results that TGs varied considerably in different types of diets, and had a high correlation with blood glucose, so it could be used as an independent factor to predict different kinds of diets. The following mechanistic diagram explained how different diets affected blood glucose and body weight by regulating changes in TGs, thereby maintaining a better health level.

## Discussion

This study showed that using refined rice and flour as a single carbohydrate source may affect body weight, blood glucose, and blood lipid levels in rats compared with the AIN-93 diet. We accurately controlled the type and amount of diet, revealed the different effects of the carbohydrate source in the diet, and determined why different dietary types affected blood glucose and body weight through lipidomics analysis. Based on the above results, we found that two TGs can independently predict the type of diet. Although a single grain of rice and flour caused an increase in blood glucose and body weight, these parameters, including TG levels, returned to normal levels after we changed the diet to an AIN-93 diet.

In the past, many studies found that dietary carbohydrate patterns could significantly change the level of blood glucose. However, most of this research compared whole grains with refined grains. Whole-grain corn powder was used to feed rats for 12 weeks in a diabetic model. The results showed that the FBG, HbAlc, TCHO, TG, LDL, and FFA contents in the experimental group were significantly reduced, glucose tolerance was significantly increased, and the degree of liver injury was reduced. These results indicated that whole grain corn powder can effectively reduce blood glucose and lipid levels (27). Some studies attribute the consumption of whole grains rich in dietary fiber to help lower blood glucose and lipid levels (28). In our research, we did not add dietary fiber or other beneficial ingredients to the control group feed. In the control group, the carbohydrates corresponding to single rice or flour were corn starch, sucrose, and maltodextrin. In our experiment, which controlled for other factors that might affect health effects, the only difference between the groups was the source of carbohydrates.

Lipidomics is an important branch of metabolomics that plays a remarkable role in nutrition research, especially in studying the effect of diet on health, obesity, and diabetes (29–33). A review detailed the methods for finding dietary biomarkers via metabolomics, which is a key technology for identifying new diet-related biomarkers (34). In this study, TGs were found to be possible biomarkers for predicting diet types that could affect health. Our study demonstrated similar results to this study, as we found two triglycerides that could better predict whether to eat flour or rice. There is a review that investigated metabolic disorders such as type 2 diabetes (T2DM), cardiovascular disease (CVD), and non-alcoholic fatty liver disease, as well as insulin resistance and obesity, which are closely related to TGs (35). Diabetes can be predicted using TGs and is related to an increased risk of diabetes, which decreases after insulin action and increases in the case of insulin resistance. These studies demonstrated how lipid analysis could contribute to clinical risk assessment for diabetes (36). The triglyceride-glucose index (TyG index) is related to T2DM and is linearly correlated with the risk of T2DM in the Japanese population, which can be used as a monitoring tool for patients (37). Calorie diet is a healthy dietary way and closely related to lipid metabolism. Caloric restriction (CR) diet can ameliorate metabolic syndrome (MetS) features, including blood lipid concentrations. The levels of TG (60:9) were negatively associated with MetS features and predicted amelioration in MetS following the intake of CR (38). The circulating TG-raising response to dietary carbohydrates in Wistar rats (39). In many studies, triglycerides have been identified as biomarkers associated with diabetes. The triglyceride markers found in our study also had a strong correlation with blood glucose levels. Predicting the type of flour diet via metabolites, which is closely related to TGs, helps distinguish differences between diets (40).

Previous studies evaluated the role of diets and were mostly performed on animals. Therefore, the potential benefits of these diets to human health should be carefully extrapolated. In our next step, we will analyze and investigate TGs and others in the people to reveal this process. The effect of bread consumption on blood glucose levels was previously studied using the gut microbiome, demonstrating a statistically significant interpersonal variability in the blood glucose response to different bread types. These results suggest that understanding dietary effects requires the integration of individual-specific factors that can also guide a personalized diet (41). Hence, using lipidomics and gut microbiome data to study the response of different diets in different people and formulate personalized diets might be a promising pathway to improve human health. There were some drawbacks to this study that we could solve in the next study. The sample size was not enough, some of our conclusions may not be fully representative of the individual differences in each rat. Even the starch structure of conspecific crops will change due to the different growth environment, varieties and processing methods. To prove potential mechanisms underlying our observations, the starch structure of the fed samples used in the three groups of rats should be measured by electron microscopy. For the problem of dosage of feed formula, we found in a similar experiment, the AIN-93 diet was used as the base formula feed for rats. They replaced sucrose in AIN-93 feed with a portion of inulin for their diet without balancing the energy and macronutrients in the diet (42). This is consistent with our design of rice and flour group feed formula. On the positive side, we retain the stability of AIN-93 as a purified feed. On the downside, we can not be clear about which specific nutrient is the most important factor influencing our results. To sum up, we have found here through a rigorous experimental design and analysis that the change of TGs as a biomarker of the health impact of dietary types. This finding provided us with a novel idea about diet and further helped to promote people to improve their diet structure.

## Conclusions

We found that single rice and flour diets resulted in significant increases in body weights and blood glucose in rats, and this adverse effect could be recovered by the AIN-93 diet. Through lipidomics, we clarified and confirmed that two TGs were closely related to the changes in blood glucose and hence could be used as an independent factor to predict the type of diet. This study recommended that a diet with multiple carbohydrate sources could help to maintain good health.

## Data Availability Statement

The original contributions presented in the study are included in the article/**Supplementary Material**, further inquiries can be directed to the corresponding authors.

## Ethics Statement

The animal study was reviewed and approved by the Ethics Committee of National Institute for Nutrition and Health, China CDC.

## Author Contributions

ZW and XX: conceptualization. MZ, BL, and ZX: data curation. SW, HM, and JW: formal analysis. XZ: methodology. WW: visualization. SW and WW: writing—original draft. SW, WW, and XX: writing—review and editing. All authors contributed to the article and approved the submitted version.

## Funding

This project was funded by the Beijing Municipal Science and Technology Commission (Z191100008619006), the Chinese Nutrition Society (CNS-ZD2019090) and the Sanming Project of Medicine in Shenzhen (SZSM201611017). The funder had no role in the study design, data collection and analysis, decision to publish, or preparation of the manuscript.

## Conflict of Interest

WW is employed by Beijing Junfeix technology Co., Ltd. The remaining authors declare that the research was conducted in the absence of any commercial or financial relationships that could be construed as a potential conflict of interest.

## Publisher's Note

All claims expressed in this article are solely those of the authors and do not necessarily represent those of their affiliated organizations, or those of the publisher, the editors and the reviewers. Any product that may be evaluated in this article, or claim that may be made by its manufacturer, is not guaranteed or endorsed by the publisher.
